# Circadian disruption decreases gluconeogenic flux in late-gestation, nonlactating dairy cows

**DOI:** 10.3168/jdsc.2022-0353

**Published:** 2023-04-28

**Authors:** Linda M. Beckett, Shawn S. Donkin, Theresa Casey

**Affiliations:** Department of Animal Sciences, Purdue University, West Lafayette, IN 47907

## Abstract

Cattle exposed to shifts in light-dark phases during late pregnancy develop hypoglycemia and insulin resistance. Our objective was to investigate if differences in liver carbon flux for gluconeogenesis were driving circadian-disrupted metabolic alterations in glucose homeostasis, and relate changes in carbon flux to hepatic gene expression. We hypothesized circadian disruption would decrease hepatic carbon flux for glucose synthesis. Milking was ceased in late-gestation Holstein cows (n = 8) at 60 d before expected calving (BEC), and animals were assigned to either a control (n = 4) or a phase-shifted (PS; n = 4) group. From d 35 to 21 BEC both groups of cows were exposed to 16 h of light and 8 h of dark, but for the PS, light was shifted forward 6 h every 3 d. On d 21 BEC, liver biopsies were collected, subdivided, and incubated in 1.0 m*M* [U-^13^C] propionate for 2 h. Total RNA was isolated from a separate liver sample and used for RNA-sequencing analysis. Postincubation ^13^C mass isotopologue distribution was determined for aspartate, serine, alanine, and glutamate and used to calculate metabolic flux ratios. Enrichment of serine to enrichment of aspartate ratio (eSer:eAsp) was lower for PS (0.75 ± 0.02) cows compared with control (0.81 ± 0.04), indicating a reduction in carbon flux toward glucose for PS animals. eSer:eAsp ratio was negatively correlated to propionyl-CoA carboxylase (*PCCB*; r = −0.79) and succinate dehydrogenase subunit D (*SDHD*; r = −0.82). These relationships indicate that when dairy cattle are exposed to circadian disruption during late gestation, propionate carbon is preferentially used for energy rather than gluconeogenesis.

## Highlights

• Dairy cattle exposed to a circadian rhythm-disrupting environment experienced altered glucose homeostasis.
• Analysis of liver tissue indicated cattle exposed to circadian disruption experienced decreased carbon flux from propionate to support glucose production.
• The relationship of carbon flow with transcriptome data indicated carbon from propionate is likely being shunted to produce energy through oxidative phosphorylation in circadian-disrupted cows rather than gluconeogenesis.

## Graphical Abstract

**Figure:**
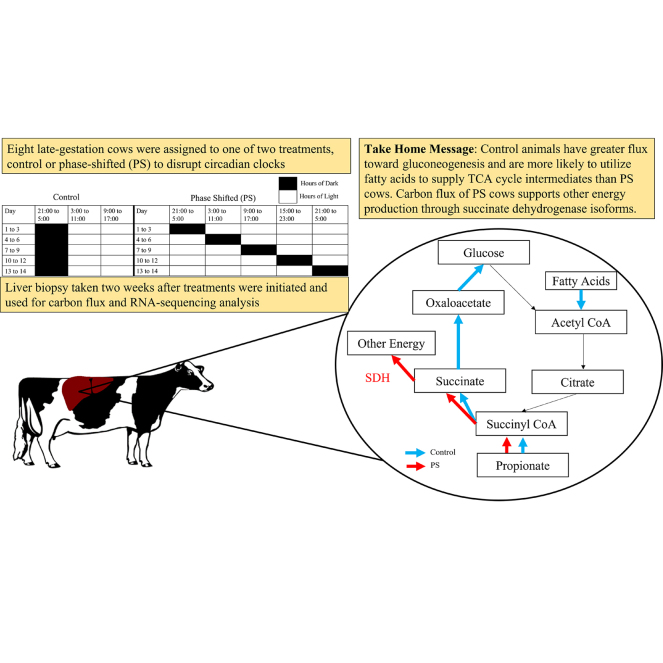
**Summary:** This study reveals cows exposed to circadian rhythm-disrupting environments decreased carbon flux from propionate to gluconeogenesis. Ruminants are dependent on gluconeogenesis for 90% of glucose needs. Therefore, dairy producers should consider effects of lighting, nutrition, and stress on circadian rhythms of dairy cattle because these may affect production efficiency, especially during times of high energetic demand. TCA = tricarboxylic acid; SDH = succinate dehydrogenase.

The high demand for glucose by the growing fetus in late gestation and to support milk production in early lactation (Bell and Bauman, 1997) challenges the ability of dairy cows to maintain glucose homeostasis. Major metabolic changes occur to support the demand for glucose, including a substantial increase in hepatic gluconeogenesis that is supported by increased uptake of propionate (Aschenbach et al., 2010). Propionate enters the tricarboxylic acid (**TCA**) cycle at succinyl-CoA as a consequence of the anaplerotic reactions initiated by mitochondrial enzyme propionyl-CoA carboxylase to form methylmalonyl-CoA. Carbon rearrangement through the actions of methylmalonyl-CoA mutase forms succinyl-CoA, which is metabolized to oxaloacetate (**OAA**) in the TCA cycle. The metabolism of OAA by phosphoenolpyruvate carboxykinase results in the formation of phosphoenolpyruvate and CO_2_ and represents the first committed step in gluconeogenesis.

Circadian clocks function as homeostatic regulators and play a central role in regulation of glucose homeostasis. In humans, circadian disruption, such as with shift work, increases the risk of obesity, hyperglycemia, hypoglycemia, and insulin resistance (Shi et al., 2019). Pregnant rats exposed to circadian rhythm–disrupting environments exhibited poor glucose tolerance and increased insulin secretion (Varcoe et al., 2011). Glucose tolerance was impaired and gestation length was protracted when pregnant sheep were exposed to a circadian disruptive environment (Gatford et al., 2019). When cows were exposed to circadian disruption through shifting of light-dark cycles starting 5 wk before calving, they developed hypoglycemia that lasted through the first weeks postpartum (Suarez-Trujillo et al., 2020). In a replicate study, cows experienced decreased insulin sensitivity as demonstrated by an intravenous glucose tolerance test, but did not develop hypoglycemia (McCabe et al., 2021). Transcriptome analysis of liver tissue from the same animals found a potential for increased development of fatty liver (Casey et al., 2021). These findings support a central role of circadian clocks in regulation of metabolism of ruminants, including factors that control glucose homeostasis. The development of hypoglycemia in Suarez-Trujillo et al. (2020) lead us to hypothesize that hepatic gluconeogenesis was impaired by circadian disruption. Our first aim was to investigate the effects of circadian disruption on hepatic carbon flux from propionate for gluconeogenesis using stable isotope flux analysis. Our second aim was to identify shifts in RNA transcripts linked to changes in hepatic carbon flux due to circadian disruption.

This study was part of a larger experiment previously described (McCabe et al., 2021) and performed in accordance with an approved protocol by Purdue University Animal Care and Use Committee (protocol no. 1701001523). Milking was ceased at d 60 before expected calving (**BEC**) in 16 multiparous, late-gestation Holstein cows. On d 35 BEC, cows were moved into tiestalls and randomly assigned to a control (n = 8) or phase-shifted (**PS**; n = 8) group. The barn was divided to separate treatments by double draping of fire-retardant tarps from floor to ceiling and black-out curtains were applied to windows to block natural light. Control cows were exposed to 16 h of light and 8 h of dark. The PS cows were also exposed to 16 h of light and 8 h of dark, but every 3 d the start of the light cycle was shifted forward 6 h. Bright white light-emitting diode (LED) lights (Smart Electrician 5000 lm 46 × 6 LED Tread Plate Shop Light, Menards Inc.) were used and the level of light at the cows' eyes was 150 lux. Cows were exposed to treatments for 14 d from d 35 BEC to d 21 BEC, thus PS cows experienced four to five 6-h shifts. Afterward, they were moved to a squeeze chute to conduct the liver biopsies on d 21 BEC.

A blind liver biopsy was performed (Greenfield et al., 2000) after clipping and cleaning an area over the 11th and 12th intercostal space with betadine and 70% alcohol. Lidocaine hydrochloride 2% (10 mL; VetOne, Midwest Animal Health) was injected, and a 1-cm incision was made. The biopsy needle was inserted 2 to 3 cm into the liver and the biopsy sample was collected under slight vacuum.

A portion of liver was immediately snap-frozen in liquid nitrogen and stored at −80°C until RNA-sequencing analysis, and approximately 250 mg was allocated toward flux analysis. The incision site was sutured closed using nonabsorbable #2 suture (Supramid). Cows were administered 10 mg/kg of ampicillin intravenously (Casey et al., 2021) and were monitored once daily for 7 d wherein sutures were removed on d 7 after biopsy.

For carbon flux analysis, liver explants (250 mg, wet weight) were immediately transported to the laboratory in 50 mL of low glucose (5 m*M*) Dulbecco's Modified Eagle Medium (**DMEM**; Fisher Scientific) containing 1% BSA (Sigma Aldrich) on ice. The liver tissue was sliced into 4 (40 mg) pieces and placed into incubation vials containing 2 mL of 5 m*M* DMEM with 1 m*M* ^12^C sodium propionate (Sigma Aldrich), 1 m*M* [U-^13^C] sodium propionate (Sigma Aldrich), 1 m*M* sodium lactate (Sigma Aldrich), 1 m*M* pyruvate, and 1% BSA. The headspace of each vial was flushed with a mixture of 95%O_2_/5% CO_2_. Vials were capped, placed in a 37°C rotating (60 rpm) water bath, and incubated for 2 h. Following incubation, liver samples were removed, blotted dry, weighed, snap-frozen in liquid nitrogen, and stored at −80°C until analysis.

Samples were extracted for GC-MS analysis as described by Kennedy and Allen (2019). Liver samples were extracted in 500 µL of 1% formic acid and 30% acetonitrile and homogenized using Precellys hard tissue homogenizing CK28 bead mill tubes (Bertin Corporation) for three 30-s periods. The 500 µL of slurry was dried in a Speedvac (Savant SPD2010 Speedvac Concentrators; Thermo Fisher Scientific). Dried samples were derivatized using 50 µL of methoxylamine hydrochloride in pyridine (2% wt/vol) and incubated at 37°C for 90 min. Then, 50 µL of MTBSTFA +1% t-BDMCS (Sigma Aldrich) was added and incubated for 30 min at 60°C (Long and Antoniewicz, 2019). Samples were centrifuged at 14,000 × *g* for 5 min at room temperature. Then, 75 µL of the supernatant was transferred to a glass GC-MS vial.

Samples were analyzed at the Purdue University Bindley Bioscience Center using the Thermo TSQ8000 Trip quadrupole MS coupled to Trace 1310 GC and Triplus RSH autosampler (Thermo Fisher Scientific) using an injection volume of 1 µL. A ThermoScientific TG-5MS column (15 m × 0.25 mm × 0.25 µm film thickness) was used for concurrent analysis of TCA cycle intermediates (not reported here) and alanine, aspartate, serine, and glutamate. The wash cycle between each sample was three 3-µL washes of acetonitrile (A) and three 3-µL washes of methanol (B). Fragments and initial retention times were obtained from Long and Antoniewicz (2019) and retention times were updated based on current GC-MS analysis. Chromeleon 7 software version 7.2 (Thermo Fisher Scientific) was used to determine area under the curve for each analyte. The area was then expressed as a mass isotopologue distribution (MID) by calculating the labeled to unlabeled ratio for each isotopologue for each analyte.

Following the protocol by El-Kadi et al. (2009), flux ratios were calculated using ^13^C tracer accumulation in select AA products. The isotope enrichment AA ratios are proxy measurements of isotope enrichment of TCA cycle and gluconeogenic intermediates assuming free exchange of carbon between these pools. Briefly, the tracer to tracee ratio was calculated for Ala, Asp, Ser, and Glu. The tracer to tracee ratio uses the ^13^C enrichment of the respective metabolite and expresses it as ^13^C [M+n] to ^12^C [M+0] enrichment. The enrichment of the specific AA pool was compared with the enrichment of [U-^13^C] propionate (99% purity). The ratio was calculated from 3 technical replicates for each animal and the average value was used for overall analysis. Ratios are calculated by comparing the sum of [M+1] to [M+3] and [M+3] only for the respective AA in that ratio. Alanine was used as a proxy for pyruvate, Glu for α-ketoglutarate (**α-KG**), Asp for OAA, and Ser for 3-phosphoglycerate (**3-PG**; Figure 1). Flux ratios were calculated for the enrichment of Ala to Asp (**eAla:eAsp**), enrichment of Glu to Asp (**eGlu:eAsp**), and enrichment of Ser to Asp (**eSer:eAsp**). The eAla:eAsp ratio characterizes the reactions from OAA through phosphoenolpyruvate and the recycling of carbon to pyruvate. The eGlu:eAsp ratio characterizes the reactions from OAA to α-KG and indicates the potential for propionate to be oxidized versus supporting gluconeogenesis. The eSer:eAsp ratio characterizes the reactions between OAA and 3-PG and describes the propensity for propionate to support gluconeogenesis versus oxidization. When the propionate purity is compared with Glu and Asp, these ratios characterize the rate of propionate oxidation to α-KG and OAA, respectively. A larger ratio is indicative of greater carbon flux from one metabolite to the other. There was poor detection of individual samples of some target AA for 4 cows (2 control and 2 PS) that had to be removed from the data set. Thus, data of 8 cows (n = 4 control and n = 4 PS) are reported here. The data have been uploaded and accepted to the Purdue University Research Repository (https://doi.org/10.4231/JB0Q-DR06).

**Figure 1:**
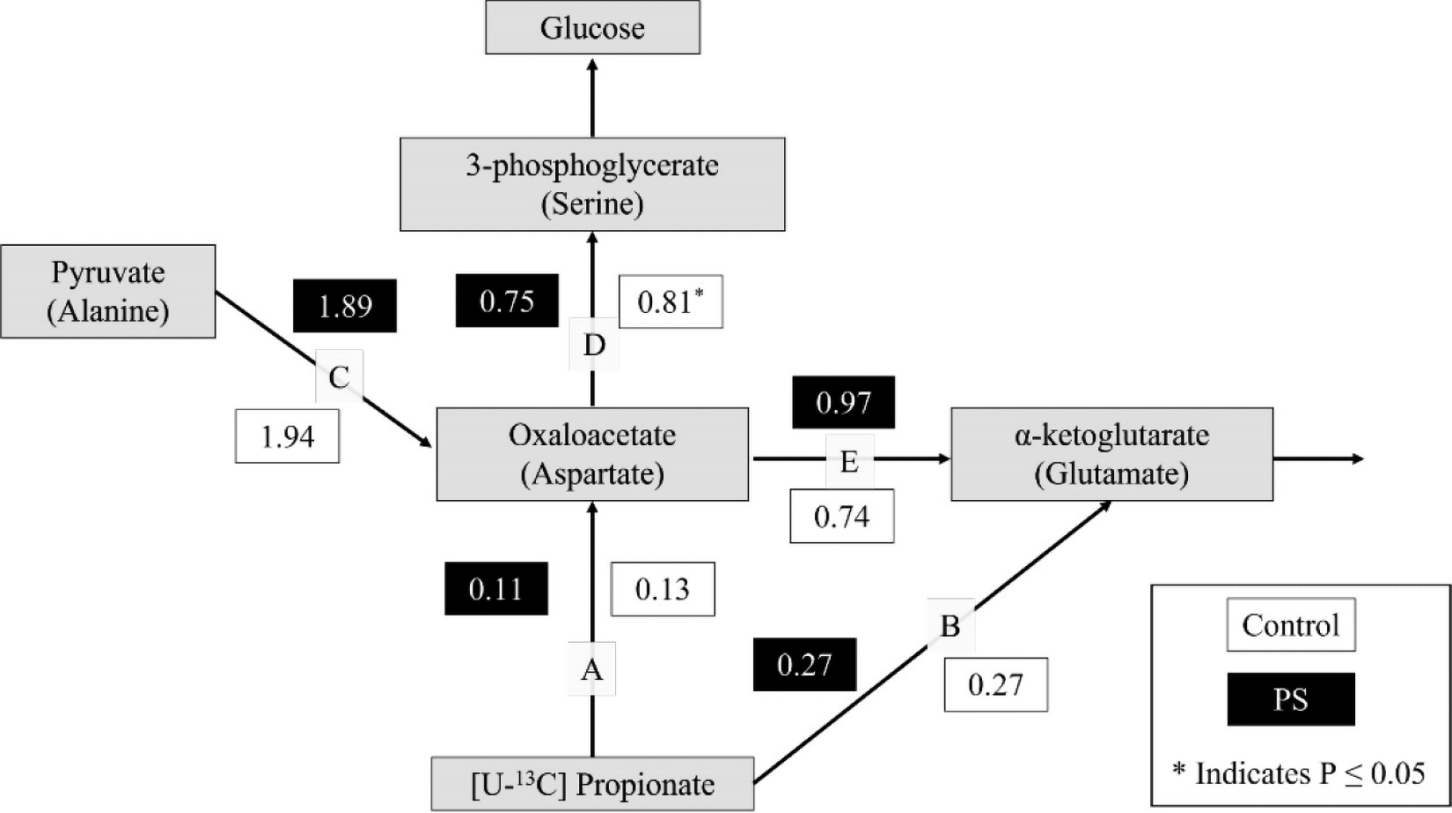
Schematic diagram representing flux of [U-^13^C] propionate to AA (shown in parentheses) that serve as proxies for the tricarboxylic acid (TCA) cycle and gluconeogenic intermediates with the assumption that there is free exchange of carbons between the AA and intermediate pools. Values represent the ratio of AA carbon enrichment in late-gestation cows exposed to control (n = 4) and phase-shifted (PS; n = 4) light-dark cycles. The eSer:eAsp ratio for control cows was significantly different (*P* = 0.05) from PS cows and represents the molecules of oxaloacetate converted to 3-phosphoglycerate, which utilizes the first committed step of gluconeogenesis. There was no significant difference between any other ratios between control and PS cows. (A) eAsp:eProp: enrichment of aspartate compared with enrichment of propionate; propionate transfer to oxaloacetate. (B) eGlu:eProp: enrichment of glutamate compared with enrichment of propionate; propionate transfer to α-ketoglutarate. (C) eAla:eAsp: enrichment of alanine compared with enrichment of aspartate; pyruvate transfer to oxaloacetate; recycling of carbons back to oxaloacetate. (D) eSer:eAsp: enrichment of serine compared with enrichment of aspartate; oxaloacetate contribution to 3-phosphoglycerate to support gluconeogenesis over TCA cycle oxidation. (E) eGlu:eAsp: enrichment of glutamate compared with enrichment of aspartate; oxaloacetate contribution to α-ketoglutarate to support continued TCA cycle oxidation over gluconeogenesis. *Significance was determined at *P* ≤ 0.05.

The RNA-sequencing analysis of liver tissue was previously described (Casey et al., 2021) and performed by GENEWIZ LLC (South Plainfield, NJ) using 50 mg of tissue from control (n = 6) and PS (n = 6) cows. The RNeasy Plus Mini Kit (Qiagen) was used to isolate total RNA, and quality was accessed with the TapeStation System and RNA ScreenTape (Agilent) (RNA integrity number score was 7.5 ± 0.4). Ribosomes were removed using QIAseq FastSelect rRNA HMR kits (Qiagen). The NEBNext Ultra II RNA Library Prep Kit for Illumina (NEB) was used to prepare the sequencing library, which was sequenced using a 2 × 150 paired-end configuration on an Illumina HiSeq 3000 RNA-sequencing instrument. Trimmomatic v.0.36 was used to remove adapter sequences and nucleotides with poor quality. The remaining trimmed reads were mapped to the bovine reference genome (ARS-UCLD1.2; https://www.ncbi.nlm.nih.gov/assembly/GCF_002263795.1/) using the STAR aligner on ENSEMBL. Unique reads were generated by using the featureCounts in the Subread package, and these reads are only present in exon regions. Differential gene expression was generated using DESeq2. Differentially expressed genes were validated in Casey et al. (2021) using real-time quantitative PCR. Data were deposited with Gene Expression Omnibus (GEO; accession number GSE168914). Despite 12 samples being sequenced, data from only 8 animals were used due to the detection limitations of the flux data.

The PROC MIXED procedure of SAS (version 9.4, SAS Institute Inc.) was used to test the effect of circadian light on the flux ratios. The model accounted for the fixed effects of phase-shift treatment and the random effects of cow within treatment. The covariance structure is autoregressive, which was determined by the lowest Akaike information criterion. Student's *t*-test was used to determine differences in means between flux ratios. Means were considered different when *P* ≤ 0.05 and tended to differ if 0.05 < *P* ≤ 0.10. To determine if differences in flux ratios were related to gene expression, linear correlation analysis was performed using the statistical package in MetaboAnalyst 5.0 (Pang et al., 2021). The resulting normalized read counts were log_2_ transformed for normality. Database for Annotation, Visualization, and Integrated Discovery (DAVID; Huang et al., 2007) was used for functional annotation analysis of genes that correlated with flux ratios. Pearson correlation coefficients for mRNA transcripts and significant flux ratios were obtained using PROC CORR (SAS Institute Inc.). In general, r-values that are found to be significant and |r| > 0.50 are sufficient to be biologically interpreted (Algina and Olejnik, 2003). To account for the smaller sample, genes discussed as being correlated with eSer:eAsp ratio had |r| > 0.70.

The eSer:eAsp ratio was lower (0.75 ± 0.02; *P* = 0.05) in PS compared with control (0.81 ± 0.04), indicating that flux of carbon from propionate to glucose was lower in PS than control cattle. There was no difference in any of the other ratios of AA enrichment (Figure 1). The lower eSer:eAsp ratio reflects a lower flux of carbons from OAA to 3-PG in PS cows relative to control cows, which indicates a reduced capacity for gluconeogenesis, which supports our hypothesis. Our hypothesis was based on the results from the Suarez-Trujillo et al. (2020) which found that PS cows developed hypoglycemia relative to control cows. However, the liver biopsies used here for flux and transcriptome analysis were taken during our replicate study (McCabe et al., 2021) where we did not observe a difference in circulating levels of glucose between control and PS cows, but found PS cows to have lower insulin sensitivity. It is possible that decreased insulin sensitivity in PS-treated cows reflected a peripheral tissue adaptation, with increased insulin resistance preventing hypoglycemia in response to lower gluconeogenic capacity of hepatic tissue. Studies of pregnant sheep conducted found exposure to circadian disruption using a chronic shift work model (alternating light-dark patterns using 12-h shifts) resulted in hypoglycemia (Varcoe et al., 2014) and decreased insulin sensitivity in response to an intravenous glucose tolerance test (Gatford et al., 2019). Circadian disruption affects glucose homeostasis at multiple molecular levels and system wide; therefore, the response at the level of the liver may be realized across the system differentially as the animal physiologically adjusts. Human and rodent response to circadian disruption spans from development of hypoglycemia (Varcoe et al., 2011) to hyperglycemia (Shi et al., 2019) and insulin resistance (Varcoe et al., 2011). Thus, differential responses in glucose homeostasis are likely due to varied and combined responses at the cellular level and systems level of the organism.

Correlation analysis indicated that eSer:eAsp varied with level of expression of 781 genes. Of the 781 genes, 366 were negatively correlated (|r| ≥ 0.70) with eSer:eAsp ratio and 415 genes were positively correlated (|r| ≥ 0.70). Ontologies most enriched with gene transcripts that negatively correlated with eSer:eAsp included protein folding, collagen, ATP binding, oxidative phosphorylation, and mitochondrial matrix/mitochondrion (Table 1). The eSer:eAsp ratio was negatively correlated with 40 genes associated with the mitochondria, the cellular location of the TCA cycle, indicating expression was relatively higher in PS than control cows. Among the gene transcripts identified were *PCCB*, *SDHAF2*, *SDHD*, *NDUFA10*, and *NDUFC2. PCCB* encodes for the β subunit of propionyl-CoA carboxylase that catalyzes the reaction of propionate to propionyl-CoA. The carbons from propionate are then metabolized to succinyl-CoA, which makes succinate and ATP. Succinate is a substrate of the complex II enzyme of the electron transport chain that mediates oxidative phosphorylation. *SDHAF2* and *SDHD* encode subunits of the succinate dehydrogenase complex and catalyze the conversion of succinate to fumarate. *NDUFA10* and *NDUFC2* encode components of NADH: ubiquinone complex I of the electron transport chain. Given a negative relationship between eSer:eAsp and *PCCB*, *SDHD*, *SDHAF2*, *NDUFA10*, and *NDUFC2* transcript abundance, it appears that carbons from propionate were being shunted toward oxidation. Thus, indicating circadian disruption increased the flow of propionate carbons toward other energy production in liver tissue.

**Table 1:** Results of functional annotation analysis of genes that positively or negatively correlated with the enrichment of serine:enrichment of aspartate (eSer:eAsp) during circadian disruption of late-gestation dairy cattle

Term^1^	Count	Total percentage of genes^2^	*P*-value^3^	Representative genes within category
Negatively correlated				
GO:0006457~protein folding	8	2.24	<0.05	*CCT6A*, *CCT8*, *ERP44*
KW-0176~collagen	4	1.12	0.05	*COL6A2*, *COL4A2*, *COLEC10*
KW-0496~Mitochondrion	20	5.60	0.04	*NDUFA10*, *HK1*, *SLC25A4*, *PCCB*, *SDHAF2*, *SDHD*, *NDUFC2*
TRANSIT: mitochondrion	10	2.80	0.06	*PCCB*, *SDHD*, *SDHAF2*, *NDUFA10*
GO:0005759~mitochondrial matrix	10	2.80	<0.05	*NDUFA10*, *PCCB*, *SDHAF2*
ATP binding	36	10.1	0.01	*HK1*, *PCCB*, *MAPKAPK2*
bta00190: oxidative phosphorylation	7	1.96	0.04	*NDUFA10*, *ATP6V1C1*, *ATP6V1E1*, *SDHD*, *NDUFC2*, *ATP6*
Positively correlated				
GO:0015485~cholesterol binding	6	1.45	<0.05	*APOA5*, *APOA4*, *APOA1*
KW-0931~ER-Golgi transport	5	1.21	0.02	*COPE*, *COPG2*, *TRAPPC2L*
KW-0496~mitochondrion	12	2.91	0.84	*ACADVL*, *NDUFS3*, *PGS1*, *NDUFA13*, *SLC25A41*

1 GO = Gene Ontology; ER = endoplasmic reticulum.

2 Total percentage of genes: the number of genes of the respective ontological category compared with the total number of genes uploaded into the Database for Annotation, Visualization, and Integrated Discovery (DAVID; Huang et al., 2007) for negatively correlated or positively correlated transcripts.

3 *P-*value: significance was determined at *P* ≤ 0.05.

Also of interest was the negative correlation of *HK1* with eSer:eAsp. Since PS animals have decreased eSer:eAsp and a negative correlation with *HK1*, this means *HK1* expression was increased in PS animals. *HK1* encodes for a hexokinase I, which converts glucose and other 6 carbon sugars to glucose-6-phosphate. Cattle hepatocytes lack functional hexokinase isozymes (Aschenbach et al., 2010). Biopsied liver tissue contains a heterogeneous population of cells including hepatocytes, Kupffer, and stellate cells. *HK1* is the most abundant hexokinase in stellate cells, and studies of mice found that activated stellate cells had lower glycolysis, but overall greater glycolytic capacity (Bae et al., 2020). Our previous analysis of the effects of circadian disruption on global transcriptome (Casey et al., 2021) indicated an increase in expression of genes activated during wound response, inflammation, and endoplasmic reticulum stress. Therefore, it is possible the stellate cells became more metabolically active because PS animals were responding to stress or inflammation thus whole-cell glycolytic activity, including expression of *HK1*, increased.

Ontologies most enriched with genes that positively correlated with the eSer:eAsp ratio included lipid transport, cholesterol binding, cholesterol efflux, positive regulation of triglyceride catabolic process, and endoplasmic reticulum-Golgi transport (Table 1). Among the 17 genes that enriched the mitochondrion ontology was acyl-CoA dehydrogenase very long chain (*ACADVL*), which encodes an enzyme that catalyzes the first step in the oxidation of very long chain fatty acids during β-oxidation. The gene encoding pyruvate dehydrogenase kinase, *PDK4*, an enzyme that catalyzes the unidirectional conversion of pyruvate to acetyl-CoA, was also in the mitochondrion ontology. Two of the most positively correlated genes (r = 0.85) were *SLC2A4* (GLUT4), an insulin-dependent glucose transporter, and *SLC5A2*, a sodium-glucose symporter.

Global changes in hepatic gene expression were also indicative of insulin-resistant states in PS cows (Casey et al., 2021). Gene expression analysis of liver before and after a hyperinsulinemic clamp found that insulin increased mRNA abundance of *ACADVL* and propionyl-CoA carboxylase α (*PCCA*) (Weber et al., 2017). Here *ACADVL* transcript abundance was positively correlated (r = 0.80; *P* = 0.015) with eSer:eAsp. The enzyme encoded by *ACADVL* catalyzes the first step of the mitochondrial fatty acid β-oxidation pathway. Acetyl-CoA, the product from β-oxidation, is oxidized in the TCA cycle and results in ATP production when coupled with oxidative phosphorylation. However, a decreased eSer:eASP with relatively lower *ACADVL* in PS animals suggests that acetyl-CoA from very long chain fatty acids is less likely to support anaplerotic reactions into the TCA cycle.

Acetyl-CoA can also be formed from pyruvate in a reaction catalyzed by pyruvate dehydrogenase (PDH). Pyruvate dehydrogenase kinase 4 (PDK4) inhibits PDH activity. Inhibition of PDH increases the availability of pyruvate for metabolism by pyruvate carboxylase to synthesize OAA. *PDK4* expression level was positively correlated with eSer:eAsp ratio. Therefore, in PS cows, the relatively lower levels of PDK4 permitted PDH to catalyze reactions that result in carbons from pyruvate to enter the TCA cycle through conversion to acetyl-CoA. The higher eSer:eAsp ratio in control cows and increased *PDK4* suggest carbons from pyruvate flow to OAA. Therefore, we surmised that in control cows relative to PS, carbon from propionate supports gluconeogenesis and carbons from fatty acids following β-oxidation support anaplerotic reactions.

The small sample size limits any strong study conclusions. Post hoc analysis found a power of 76.5% regarding eSer:eAsp difference between groups. The assumptions of equal labeling of all pools and free exchange of carbon between the metabolite pools may also be a study limitation. Further research should measure TCA cycle and gluconeogenic intermediates and possibly use a different ^13^C tracer, such as palmitic acid, which could provide more information into the level of saturation of different pools like acetyl-CoA. Nonetheless, the data do indicate that hepatic gluconeogenesis is impaired in cows exposed to circadian-disrupting environments, and are consistent with studies of other species.

In conclusion, carbon flux analysis indicated that circadian rhythm disruption in late-gestation cows impairs hepatic gluconeogenesis. Analysis of the relationship between carbon flux and transcript abundance indicated that cows experiencing circadian disruption will preferentially use carbon from propionate for energy production rather than glucose synthesis. Producers should consider light, nutrition, stress, reproductive status, and other factors that may affect circadian rhythms and affect production efficiency of cattle especially when there is a high demand for glucose from gluconeogenesis.
